# The physical health and premature mortality of Indigenous Māori following first-episode psychosis diagnosis: A 15-year follow-up study

**DOI:** 10.1177/00048674241270981

**Published:** 2024-08-21

**Authors:** Nathan J Monk, Ruth Cunningham, James Stanley, Sue Crengle, Julie Fitzjohn, Melissa Kerdemelidis, Helen Lockett, Andre D McLachlan, Waikaremoana Waitoki, Cameron Lacey

**Affiliations:** 1 Department of Māori/Indigenous Health Innovation, University of Otago, Christchurch, New Zealand; 2 Department of Public Health, University of Otago, Wellington, New Zealand; 3 Ngāi Tahu Māori Health Research Unit, Division of Health Sciences, University of Otago, Christchurch, New Zealand; 4 Specialist Mental Health Service, Te Whatu Ora – Waitaha Canterbury, Christchurch, New Zealand; 5 Population Health Gain, Service Improvement and Innovation, Te Whatu Ora – Waitaha Canterbury, Christchurch, New Zealand; 6 Te Pou, Wellington, New Zealand; 7 Centre for Health and Social Practice, Waikato Institute of Technology, Hamilton, New Zealand; 8 Faculty of Māori and Indigenous Studies, The University of Waikato, Hamilton, New Zealand; 9 Department of Psychological Medicine, University of Otago, Christchurch, New Zealand

**Keywords:** First-episode psychosis, New Zealand, Māori, indigenous health, health equity, physical health, cardiometabolic health, premature mortality, cohort study

## Abstract

**Background:**

People experiencing psychosis are at greater risk of physical health conditions and premature mortality. It is likely that Indigenous Māori youth, who experience additional systemic inequities caused by settler-colonisation, face even greater physical health and mortality risks following a diagnosis of first-episode psychosis.

**Objective:**

Compare Māori and non-Māori for risk of hospitalisation and mortality for up to 15 years following first-episode psychosis diagnosis.

**Methods:**

A cohort (*N* = 14,122) of young people (16–24 years) with first-episode psychosis diagnosis between 2001 and 2019 were identified. Using crude Kaplan–Meier and adjusted Cox proportional hazards models, Māori (*n* = 5211) and non-Māori (*n* = 8911) were compared on hospitalisation and mortality outcomes for up to 15 years.

**Results:**

In the 15 years following first-episode psychosis diagnosis, Māori had higher adjusted risk of all-cause mortality (hazard ratio = 1.21, 95% confidence interval = [1.01, 1.45]), hospitalisation with diabetes (hazard ratio = 1.44, 95% confidence interval = [1.15, 1.79]), injury/poisoning (hazard ratio = 1.11, 95% confidence interval = [1.05, 1.16]), general physical health conditions (hazard ratio = 1.07, 95% confidence interval = [1.02, 1.13]) and also appeared to be at greater risk of cardiovascular hospitalisations (hazard ratio = 1.34, 95% confidence interval = [0.97, 1.86]). Kaplan–Meier plots show hospitalisation and mortality inequities emerging approximately 4–7 years following first-episode psychosis diagnosis.

**Conclusions:**

Māori are at greater risk for hospitalisation and premature mortality outcomes following first-episode psychosis. Early screening and intervention, facilitated by culturally safe health service delivery, is needed to target these inequities early.

## Background

Psychosis is a feature of some serious mental illness, such as schizophrenia and type I bipolar disorder (Firth et al., 2019). People with psychosis report a wide range of experiences, including symptoms related to impaired perception of reality. Some commonly reported psychosis symptoms include delusions, hallucinations, paranoia, grandiose ideation and loss of agency or control (Fusar-Poli et al., 2022). Both worldwide and in Aotearoa New Zealand (NZ), people experiencing psychosis have premature mortality, living, on average, approximately 20 years less than the general population (Cunningham et al., 2014; Laursen et al., 2014; Te Pou o Te Whakaaro Nui, 2014; Walker et al., 2015). Premature mortality for people experiencing psychosis is primarily driven by premature deaths from physical illness, injury/poisoning and suicide (Correll et al., 2017; Cunningham et al., 2014; Firth et al., 2019; Olfson et al., 2015).

There are many systemic contributors to physical health inequity for people experiencing psychosis. Relative to the general population, they are more often exposed to common risk factors for long-term physical health conditions (e.g. smoking, problematic alcohol use, poor access to nutrition and sedentary behaviour), such as cardiovascular disease (CVD) and diabetes (Di Florio et al., 2014; Jackson et al., 2015; Koskinen et al., 2009; Quigley and MacCabe, 2019; Stubbs et al., 2016; Teasdale et al., 2019; Vancampfort et al., 2017). Notably, risk of CVD and diabetes are increased by cardiometabolic side effects of commonly prescribed second-generation antipsychotic medications (Galletly et al., 2012, 2016; Mitchell et al., 2011). People experiencing psychosis also typically have reduced access to physical health care, and sub-optimal quality of care (Ayerbe et al., 2018; Cunningham et al., 2023a, 2023b; Firth et al., 2019).

It is also important to consider how disadvantages related to psychosis may be compounded for people also experiencing systemic racism. The experience of multiple systemic contributors to physical ill health (i.e. psychosis and racism, in this case) may produce a cumulative detriment to health for people in these communities (Denise, 2014; Dowd and Bengtson, 1978). When considering the intersection of psychosis and ethnicity, diagnoses of psychotic disorders have been reported as more common in non-dominant ethnicities across many settings (Gynther et al., 2019; Jongsma et al., 2018; Schofield et al., 2016; Termorshuizen et al., 2014; Veling, 2013). Empirical studies across these various settings have yielded mixed findings when comparing the physical health of dominant and non-dominant ethnic groups experiencing psychosis (e.g. Cunningham et al., 2020; Das-Munshi et al., 2017; Daumit et al., 2010; Musuuza et al., 2013).

In NZ, Indigenous Māori experience gross health inequity when compared to non-Māori, and particularly when compared to NZ Europeans (Ministry of Health, 2018, 2019). Māori health inequity is understood as an outcome of historical and contemporary settler-colonisation, which has entrenched systemic racism (Lacey et al., 2022; Moewaka Barnes and McCreanor, 2019; Reid et al., 2019). Due to systemic advantages for primarily NZ European people, Māori are more likely than non-Māori to experience determinants of poor health, such as socioeconomic deprivation and other forms of racism (Harris et al., 2018; Ministry of Health, 2019, 2023). Against this background, Māori experience higher rates of long-term physical health conditions, such as CVD, diabetes and cancer, and are also more likely to be diagnosed with psychotic disorders (Kake et al., 2008; Linscott et al., 2006; Ministry of Health, 2019, 2023; Petrović-van der Deen et al., 2020). Due to reported barriers, Māori have poorer access to health services, leading to worse outcomes from physical health conditions when compared to non-Māori (Espiner et al., 2021; Graham and Masters-Awatere, 2020; Jansen et al., 2009; Ministry of Health, 2019, 2023). Moreover, Māori-run service providers have been strategically underfunded by the Crown (Waitangi Tribunal, 2021). Consequently, Māori have a life expectancy approximately 7 years less than non-Māori (Ministry of Health, 2018).

First-episode psychosis (FEP) is the first clinical presentation of psychosis, and may be the onset of psychotic disorder, such as schizophrenia. Age of FEP onset is typically adolescence or early adulthood (Reed, 2008; Solmi et al., 2022a). Despite relatively early onset, several longitudinal studies have shown that the risk of premature mortality, from both physical illness and other causes, increases substantially following FEP diagnosis (Craig et al., 2006; Schoenbaum et al., 2017; Simon et al., 2018; Tanskanen et al., 2018; Yung et al., 2023). In NZ, rangatahi (young) Māori are approximately twice as likely as non-Māori youth to be diagnosed with FEP (Petrović-van der Deen et al., 2020). Given the increase in physical morbidity and mortality risk associated with psychosis, this is a considerable health equity concern.

## Te Pūrākau o Te Pu Korokoro

This study is from Phase 1 of a larger research project, *Te Pu Korokoro: Improving the physical health of Māori with psychosis*. In May each year, the kuaka (godwit) birds migrate 12,000 km from Aotearoa (NZ) to Alaska. In September, the kuaka arrive back in Aotearoa from Alaska. They settle along the mudflats in Tāmaki (Auckland), and for over a 1000 km down the east coast to Ōtautahi (Christchurch). *Te Pu Korokoro* symbolises the air pocket formed during the kuaka’s flight; it is a place to rest and be protected from the winds. *Tāngata whaiora* (people seeking wellness) and their supporters describe falling through gaps and losing support, like falling from an air pocket into harsh winds. Like the migration cycle of the kuaka, *Te Pu Korokoro* has several phases, and all are essential. This paper is the first in a series that aims to generate knowledge to improve outcomes, and ultimately deliver recommendations based on the voices of Māori experiencing psychosis.

## Aims

This study analyses the physical health of a cohort of young people for up to 15 years following FEP diagnosis. In particular, rates of hospitalisation and premature mortality are compared between Māori and non-Māori. Within hospitalisations, we focused particularly on CVD- and diabetes-related admissions, as major causes of morbidity associated with psychosis diagnoses. The aims of this study are:

1. Estimate and compare the risk of hospitalisation events (including CVD- and diabetes-specific admissions) in Māori and non-Māori for up to 15 years following FEP diagnosis.
2. Estimate and compare the risk of premature mortality (all-cause and cause-specific) in Māori and non-Māori for up to 15 years following FEP diagnosis.

## Method

### Ethics

The authors assert that all procedures contributing to this work comply with the ethical standards of the relevant national and institutional committees on human experimentation and with the Helsinki Declaration of 1975, as revised in 2008. All procedures involving human subjects/patients were approved by University of Otago Ethics Committee, reference number HD22/089.

### Participants

Two datasets from Te Whatu Ora/Health New Zealand National Collections were used to obtain a cohort of people with a diagnosis of FEP. The Programme for the Integration of Mental Health Data (PRIMHD) contains all contacts with secondary mental health and addiction services since July 2007 (Te Whatu Ora/Health New Zealand, 2023e). The National Minimum Dataset (NMDS) contains all public and many private hospitalisation data (Te Whatu Ora/Health New Zealand, 2023c). Encrypted National Health Index (NHI) identifiers were used to link patient records between all National Collections datasets used in this study. Participant consent is not required to analyse de-identified National Collections records (Te Whatu Ora/Health New Zealand, 2023a).

People with a Diagnostic and Statistical Manual of Mental Disorders (4th ed.; *DSM*-IV), International Classification of Diseases, Ninth Revision (ICD-9) or ICD-10 diagnostic code (principal, other relevant or provisional) recorded for a psychotic disorder between 2001 and 2019 in either PRIMHD or the NMDS were included, covering diagnoses associated with both inpatient and outpatient interactions. Diagnostic codes include schizophrenia, bipolar disorder, schizoaffective disorder, depressive disorder with psychotic symptoms, organic psychotic disorder, substance-induced psychotic disorder and psychosis not otherwise specified (NOS; see Supplementary Table S1 for codes).

Previous hospitalisation records were checked back to 1997 to rule out prior psychosis diagnosis. People aged 16–24 years at FEP diagnosis were retained in the cohort. For each person, a maximum of 15 years of follow-up data were extracted, meaning the oldest people (those aged 24 years at FEP diagnosis, with 15 years of follow-up data available) were 39 years old at the end of follow-up. The date of each person’s first recorded psychosis diagnosis (in either PRIMHD or NMDS) was taken as their index date for follow-up data extraction.

### Measures

#### Ethnicity

Reported analyses compare rangatahi Māori to non-Māori youth (all aged 16–24 years at FEP diagnosis). Ethnicity is recorded in the Ministry of Health’s NHI dataset, and should be collected and checked at recommended intervals during healthcare interactions, with protocols for collection stipulating that this should be self-identified ethnicity (Health Information Standards Organisation, 2017). Māori were identified by prioritised ethnicity; people were included in the rangatahi Māori cohort if they self-identified as Māori, regardless of any other ethnicities. All other people were categorised as non-Māori.

#### Physical health measures

Records for hospitalisation (NMDS), pharmaceutical dispensing (Te Whatu Ora/Health New Zealand, 2023d) and mortality (Te Whatu Ora/Health New Zealand, 2023b) were linked to each person anonymously via encrypted NHI number.

#### Physical health status at FEP diagnosis (M3)

Physical health status at FEP diagnosis was assessed using a modified version of the M3 multimorbidity index (Stanley and Sarfati, 2017), which quantifies multimorbidity using ICD-10 diagnoses recorded in hospitalisation data over a 5-year lookback from index date (FEP diagnosis for the present cohort), and assigns weights from 55 medical conditions based on their predictiveness of mortality. For this study, five psychiatric categories were excluded from the M3 score calculation (major psychiatric disorder; mental and behavioural disorder; anxiety and behavioural disorder; substance use disorder) to focus on physical conditions. To describe physical health at FEP diagnosis, M3 scores were categorised into 0, > 0 to < 1, 1 to < 2, and ⩾ 2. A score of zero indicates that no relevant diagnostic codes were present in hospitalisation data; higher scores indicate presence of condition(s) associated with higher mortality.

#### Physical health following diagnosis

Following index date (FEP diagnosis), up to 15 years of follow-up health data were extracted for each person, up to 31 October 2021. Hospitalisation and mortality records were used to define the main outcomes following FEP diagnosis. All hospitalisations were filtered to only include medical and surgical hospital admissions (Health Specialty Codes beginning ‘M’ or ‘S’), hence excluding psychiatric unit admissions.

##### Physical health hospitalisations

Physical health hospitalisations were defined as hospitalisation events where there was a recorded diagnosis (primary or secondary) of a physical health condition (ICD-9 codes 001–289, 320–629, 680–739; ICD-10 codes A00–E89, G00–N99). Thus, hospitalisations with only psychiatric diagnoses were excluded. Admissions containing only diagnoses relating to pregnancy and childbirth (e.g. gestational diabetes) were also excluded to avoid bias, as Māori have a mean maternal age 3.5 years younger than non-Māori, and are more likely than non-Māori to have children before age 20 years (Marie et al., 2011; Rarere et al., 2023).

##### Injury/poisoning hospitalisations

Injury/poisoning hospitalisations were defined as hospitalisation events where any injury/poisoning (including burns) diagnosis (primary or secondary) was recorded (ICD-9 codes 800–999; ICD-10 codes S00–T88).

##### Diabetes hospitalisations

Diabetes hospitalisations included hospitalisations where any diagnosis (primary or secondary) of diabetes was recorded (ICD-9 codes 249 and 250; ICD-10 codes E08–E13), excluding gestational diabetes.

##### CVD hospitalisations

CVD hospitalisations included hospitalisations where any diagnosis (primary or secondary) of CVD was recorded. Categories included from both ICD-9 and ICD-10 codes were: myocardial infarction, unstable angina, ischaemic stroke, haemorrhagic stroke, transient ischaemic attack, other coronary heart disease, peripheral vascular disease, congestive heart failure, and other ischaemic CVD-related codes (see Supplementary Table S2 for codes).

##### All-cause mortality, non-medical mortality and medical mortality

Premature mortality was categorised into all-cause mortality (death from any cause), non-medical mortality (intentional and accidental events of ‘external cause’ resulting in injury, poisoning or burns; ICD-10 codes starting with V, W, X or Y as underlying cause of death) and medical mortality (any other ICD-10 code as underlying cause of death).

#### Covariates

Gender and date of birth were obtained from the Ministry of Health NHI dataset. Socioeconomic deprivation was assessed with NZDep2018 quintiles, which indicates the relative deprivation for small geographic areas of NZ based on the 2018 NZ census (Atkinson et al., 2019).

Non-psychotic psychiatric comorbidities were identified from the NMDS and PRIMHD, and were categorised into depression, anxiety, substance use disorders and personality disorders. In NZ, for inpatient admissions, diagnostic coding is performed by trained coders who code each diagnosis based on a standardised set of rules applied to the clinical file. In outpatient specialist care, a diagnosis must be entered by clinicians either at discharge or after 3 months of care.

### Analysis

All data cleaning and statistical analysis was performed in R (R 4.1, R Institute, Vienna, Austria).

Descriptive analyses present demographic characteristics, physical multimorbidity at index date and other clinical characteristics for Māori and non-Māori. These descriptive analyses are presented as profiles (counts and percentages): no formal statistical comparisons are made, as the objective of this table is to describe general differences by ethnicity that may be relevant for considering confounding (Vandenbroucke et al., 2007).

To handle variable follow-up periods, crude Kaplan–Meier analysis was performed to estimate the risk of outcome events occurring over a maximum of 15 years following FEP diagnosis. Separate Kaplan–Meier functions compared Māori and non-Māori for each outcome event: all-cause mortality, medical mortality, non-medical mortality, physical health hospitalisation, injury/poisoning hospitalisation, diabetes hospitalisation and CVD hospitalisation. In analyses of hospitalisation outcomes, records were censored at death if it occurred before hospitalisations were observed. Records were also censored at death in cause-specific mortality plots if the person died of another cause.

Further Kaplan–Meier analysis was performed to separate injury/poisoning hospitalisation and non-medical mortality categories into intentional (self-harm/suicide) and presumed accidental events. Injury/poisoning hospitalisations were coded as self-harm if any self-harm ICD-9 or ICD-10 code was recorded for the event; all other injury/poisoning hospitalisations were coded as accidental. Non-medical deaths were coded as suicide if any self-harm/suicide ICD-10 code was recorded for the death; all other non-medical deaths were coded as accidental.

Cox proportional hazards regressions were performed to adjust for confounding while allowing for censoring of follow-up time. Crude and adjusted (age, gender and socioeconomic deprivation) Cox regression models estimate the risk of follow-up events for Māori relative to non-Māori. Supplementary Cox regression analyses were performed to explore gender differences in Māori and non-Māori.

## Results

A total of 14,122 people were diagnosed with FEP between 2001 and 2019 while aged 16–24 years. Of these, 9074 FEP diagnoses were recorded in hospital admissions data (NMDS), and an additional 5048 were identified from mental health service data (PRIMHD).^1^ Of this total cohort, 5211 (36.9%) were rangatahi Māori. All analyses compare this rangatahi Māori cohort (*n* = 5211) to non-Māori youth (*n* = 8911).

Table 1 shows the demographic and clinical characteristics for rangatahi Māori and non-Māori youth. Māori were younger at FEP diagnosis, and had higher exposure to socioeconomic deprivation, with 46.7% of Māori living in areas in the most deprived quintile, and 73.5% in the two most deprived quintiles. Over the study (including FEP and follow-up diagnoses), Māori were more likely to receive diagnoses of schizophrenia, schizoaffective disorder, substance-induced psychosis, organic psychosis, other psychosis and psychosis NOS. Non-Māori were more likely to receive diagnoses of bipolar disorder and depression with psychosis, and were more likely to be solely diagnosed with psychosis NOS. In terms of non-psychotic psychiatric comorbidities, Māori were more likely to be diagnosed with a substance use disorder, and less likely than non-Māori to be diagnosed with depression or an anxiety disorder. Both Māori and non-Māori had very low physical multimorbidity (as measured by hospitalisation diagnoses) at the time of FEP diagnosis. Over 90% of both Māori (91.9%) and non-Māori (91.8%) did not receive any of the M3 index physical health diagnoses in an inpatient hospital setting in the 5 years prior to FEP diagnosis.

**Table 1.** Cohort demographic and clinical characteristics compared between Māori (*n* = 5211) and non-Māori (*n* = 8911) who experienced FEP, at index date and in 15-year follow-up.

		Māori	Non-Māori
		*n* (%)	*n* (%)
Total *N*		5211	8911
Gender^a^	Male	3376 (64.8)	5522 (62.0)
	Female	1833 (35.2)	3379 (37.9)
Age at index	Mean	19.9	20.4
	16–18 years	1679	2233
	19–21 years	1993	3532
	22–24 years	1539	3146
Deprivation quintile	1	250 (4.8)	1123 (12.6)
	2	441 (8.5)	1468 (16.5)
	3	670 (12.9)	1685 (18.9)
	4	1398 (26.8)	2433 (27.3)
	5	2431 (46.7)	2081 (23.4)
Entry FEP diagnosis	Schizophrenia	1062 (20.4)	1334 (15.0)
	Bipolar	702 (13.5)	2133 (23.9)
	Depressive	330 (6.3)	807 (9.1)
	Substance	852 (16.4)	954 (10.7)
	Schizoaffective	45 (0.86)	67 (0.75)
	Other	579 (11.1)	883 (9.9)
	Organic	13 (0.25)	24 (0.27)
	Psychosis NOS	1628 (31.2)	2709 (30.4)
All psychosis diagnoses^b^	Schizophrenia	2751 (52.8)	3182 (35.7)
	Bipolar	1199 (23.0)	2918 (32.8)
	Depressive	465 (8.9)	1066 (12.0)
	Substance	1573 (30.2)	1632 (18.3)
	Schizoaffective	798 (15.3)	837 (9.4)
	Other	1171 (22.5)	1695 (19.0)
	Organic	67 (1.3)	80 (0.9)
	Psychosis NOS	2690 (51.6)	4115 (46.2)
	Psychosis NOS only	556 (10.7)	1182 (13.3)
Other psychiatric diagnoses	Substance	3540 (68.1)	4060 (45.7)
	Depression	835 (16.1)	1931 (21.8)
	Anxiety	539 (10.6)	1623 (18.4)
	Personality	834 (16.3)	1360 (15.4)
Physical multimorbidity at index	Mean M3 score	0.043	0.045
	0	4790 (91.9)	8181 (91.8)
	> 0 to < 1	367 (7.04)	610 (6.85)
	1 to < 2	45 (0.86)	99 (1.11)
	⩾ 2	9 (0.17)	21 (0.24)
Follow-up time in years	Median (IQR)	9 (5–14)	10 (6–14)

FEP: first-episode psychosis; NOS: not otherwise specified; IQR: interquartile range.

a A total of 12 people were recorded as other or unknown gender.

b Diagnoses are included from entire study period including follow-up (apart from FEP diagnosis).

Table 2 shows the risk of follow-up events for Māori and non-Māori within 15 years of follow-up. The median follow-up time was 9 years (interquartile range [IQR] = 5–14) for Māori and 10 years (IQR = 6–14) for non-Māori. Visual inspection of Kaplan–Meier log–log transformation plots indicated that the proportional hazards assumption was acceptable for hospitalisation and mortality outcomes. Māori had higher risk of all measured outcomes compared to non-Māori at the end of follow-up. After adjusting for confounding (age, gender and socioeconomic deprivation), Māori had greater risk of all-cause mortality (hazard ratio [HR] = 1.21, 95% confidence interval [CI] = [1.01, 1.45]), physical health hospitalisation (HR = 1.07, 95% CI = [1.02, 1.13]), injury/poisoning hospitalisation (HR = 1.11, 95% CI = [1.05, 1.16]) and diabetes hospitalisation (HR = 1.44, 95% CI = [1.15, 1.79]). There was also some indication that Māori were at greater risk of CVD hospitalisation; however, this finding did not exclude the null (HR = 1.34, 95% CI = [0.97, 1.86]). Supplementary analysis showed that significant gender differences in outcome risk were the same for Māori and non-Māori: females were at greater risk of physical health, injury/poisoning and diabetes hospitalisations; males were at greater risk of all-cause mortality (see Supplementary Table S3).

**Table 2.** Follow-up mortality and hospitalisation risk and Cox regression hazards for Māori (*n* = 5211) compared to non-Māori (*n* = 8911) experiencing psychosis.

	Kaplan–Meier risk estimates for event by end of follow-up period (15 years)		Cox proportional hazards model	
	Māori *n* events (Kaplan–Meier risk estimate)	Non-Māori *n* events (Kaplan–Meier risk estimate)	Crude HR (95% CI)	Adjusted HR^a^ (95% CI)
Mortality, all cause^b^	217 (6.5%)	302 (4.9%)	1.24 [1.04, 1.48]	1.21 [1.01, 1.45]
Mortality, medical cause	32 (1.2%)	47 (0.9%)	1.18 [0.75, 1.85]	1.13 [0.71, 1.81]
Mortality, non-medical cause	135 (3.8%)	195 (3.0%)	1.19 [0.96, 1.49]	1.14 [0.90, 1.43]
Hospitalisation, physical health	2364 (59.2%)	3821 (54.0%)	1.09 [1.03, 1.14]	1.07 [1.02, 1.13]
Hospitalisation, injury/poisoning	2614 (65.5%)	4091 (57.6%)	1.13 [1.07, 1.19]	1.11 [1.05, 1.16]
Hospitalisation, diabetes	165 (6.1%)	183 (3.3%)	1.57 [1.27, 1.94]	1.44 [1.15, 1.79]
Hospitalisation, CVD	70 (2.5%)	91 (1.7%)	1.33 [0.97, 1.82]	1.34 [0.97, 1.86]

HR: hazard ratio; CI: confidence interval; CVD: cardiovascular disease.

a Adjusted for age, gender and socioeconomic deprivation.

b Cause-specific deaths do not sum to total deaths because cause-of-death data were not available for all recorded deaths.

Further analysis was performed to separate injury/poisoning hospitalisation and non-medical mortality categories into self-harm/suicide and presumed accidental events. This analysis is reported in Table 3, with the risk of each event type for Māori and non-Māori. Māori had higher risk of accidental injury/poisoning hospitalisation (*p* < 0.001) and accidental death (*p* = 0.013) than non-Māori. However, suicide was the most common cause of death for both Māori and non-Māori (210/409 of all deaths with a recorded cause). Accidental poisoning (*n* = 44) and transport accidents (*n* = 37) accounted for 81/120 (67.5%) accidental deaths. Of the 44 accidental poisoning deaths, 22 were primarily due to medications and 22 were primarily due to narcotics, alcohol and other substances.^2^

**Table 3.** Kaplan–Meier risk estimates for 15-year follow-up injury/poisoning hospitalisations and non-medical deaths by recorded intentionality, for Māori (*n* = 5211) and non-Māori (*n* = 8911).

		Māori *n* events (Kaplan–Meier risk estimate)	Non-Māori *n* events (Kaplan–Meier risk estimate)	Chi-square *p*-value
Injury/poisoning hospitalisations				
	Self-harm	687 (16.5%)	1290 (17.3%)	0.044
	Accidental	2358 (61.2%)	3577 (52.6%)	< 0.001
Non-medical deaths				
	Suicide	78 (2.1%)	132 (1.9%)	0.898
	Accidental	57 (1.7%)	63 (1.0%)	0.013

Hospitalisations sum to greater than total injury/poisoning hospitalisations (see Table 2) as people can experience both self-harm and accidental hospitalisations in separate events during follow-up; deaths sum to total non-medical deaths (see Table 2), as death occurs only once.

**Figure 1.**
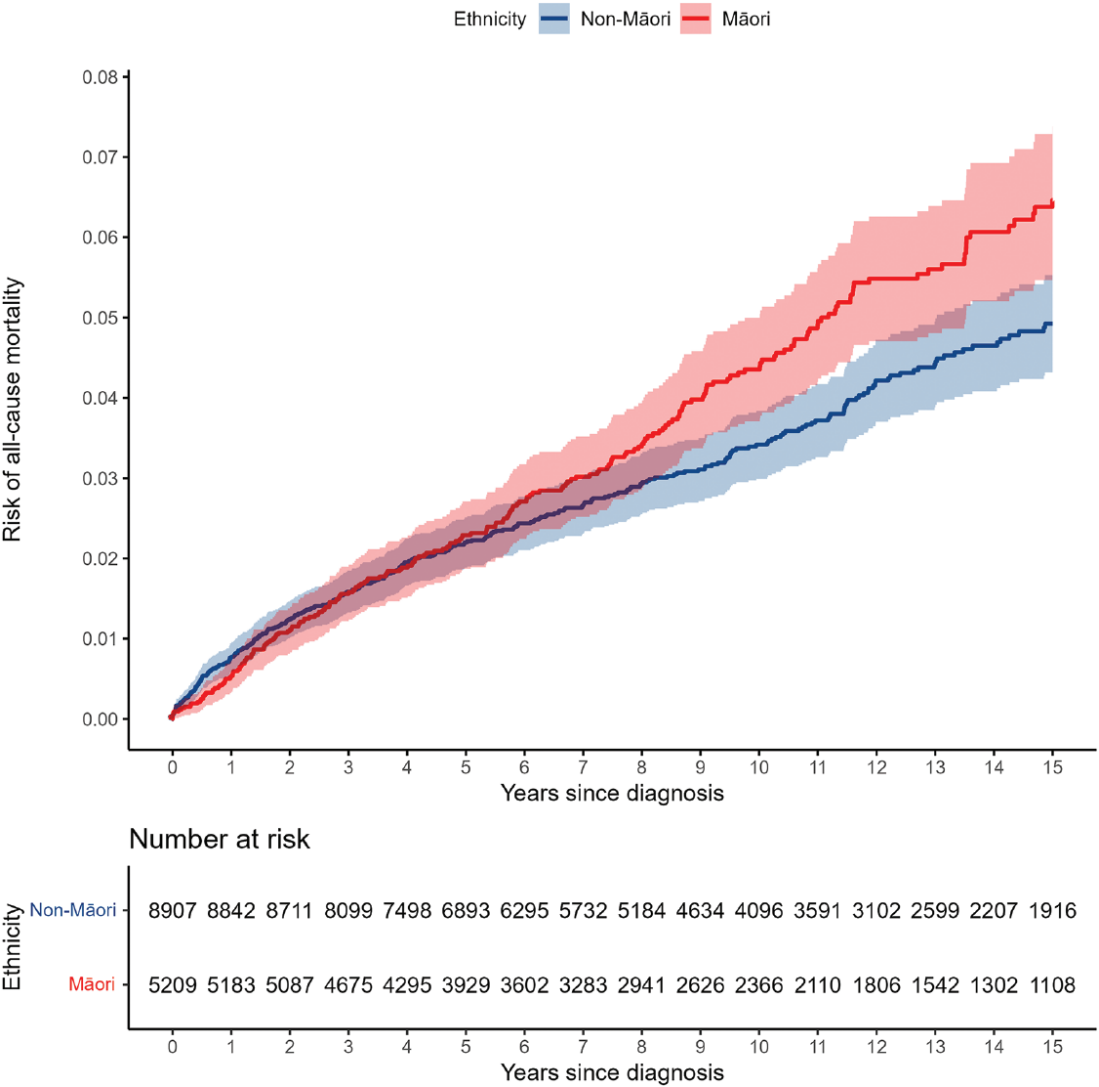
Māori and non-Māori Kaplan–Meier risk of all-cause mortality in 15-year follow-up from first-episode psychosis (shaded areas indicate 95% confidence intervals).

**Figure 2.**
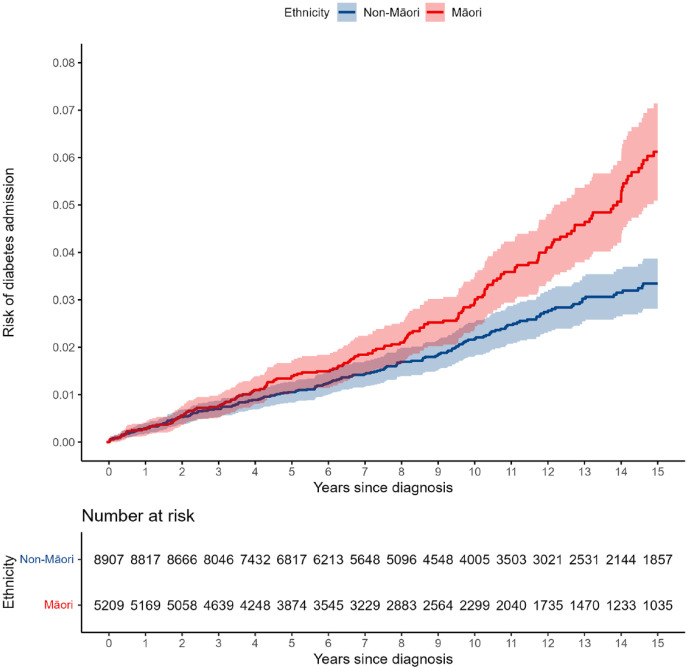
Māori and non-Māori Kaplan–Meier risk of diabetes hospitalisation in 15-year follow-up from first-episode psychosis (shaded areas indicate 95% confidence intervals).

**Figure 3.**
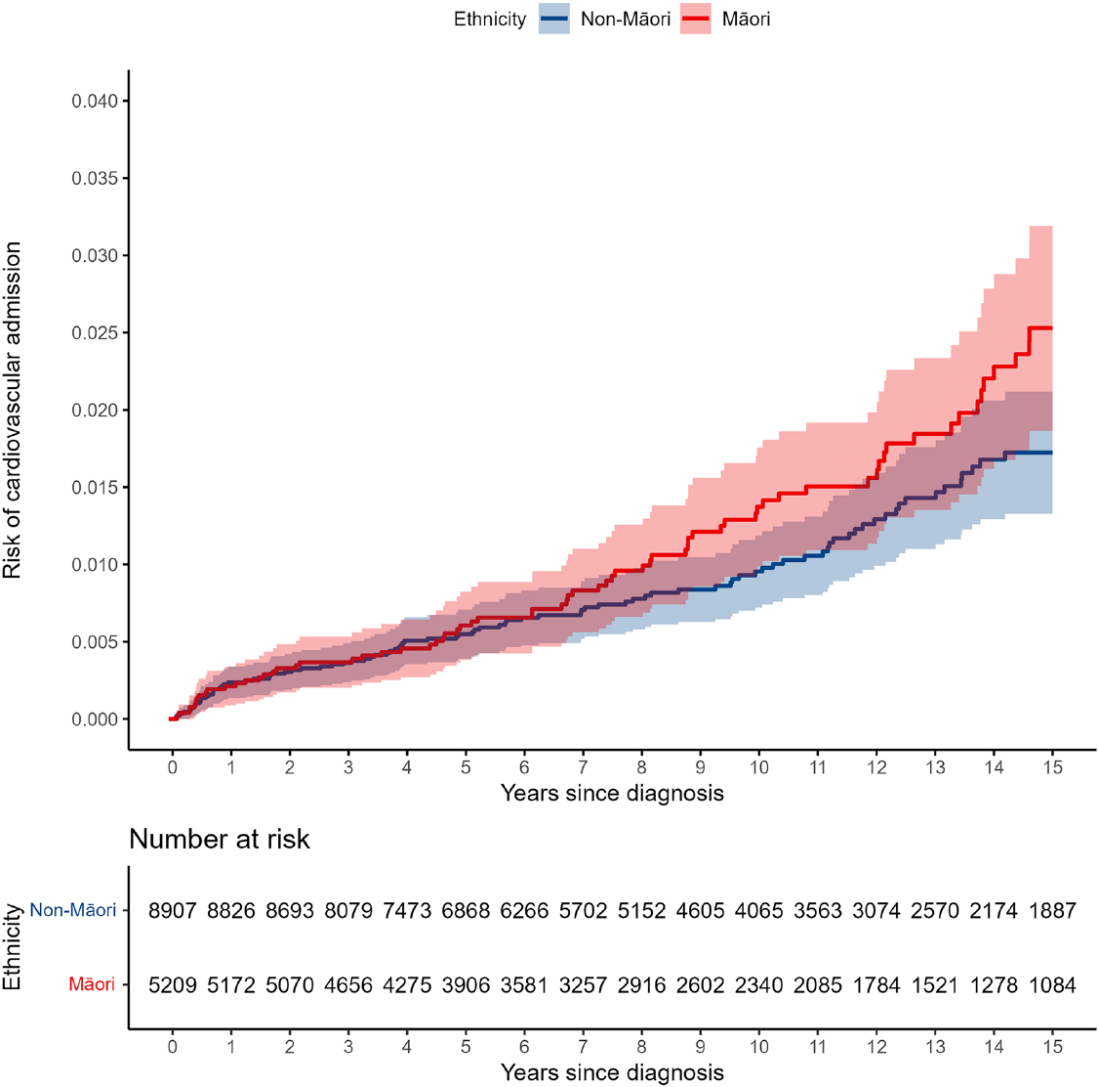
Māori and non-Māori Kaplan–Meier risk of cardiovascular diseases hospitalisation in 15-year follow-up from first-episode psychosis (shaded areas indicate 95% confidence intervals).

The Kaplan–Meier plots for all-cause mortality (Figure 1) diabetes hospitalisation (Figure 2) and CVD hospitalisation (Figure 3) are presented, comparing the crude Kaplan–Meier estimates for Māori and non-Māori over the follow-up period. Kaplan–Meier plots for other study outcomes are included in Supplementary materials (see Figures S1–S4). For outcomes with differences between Māori and non-Māori, the Kaplan–Meier functions for Māori and non-Māori appear to begin diverging approximately 4–7 years after FEP diagnosis, indicating physical health inequities for Māori experiencing psychosis begin to emerge in these data during this period.

## Discussion

### Summary and interpretation of results

We analysed the hospitalisation and mortality records of 14,122 people for up to 15 years following FEP diagnosis at 16–24 years old. Māori had a higher adjusted risk of all-cause mortality, physical health hospitalisation, injury/poisoning hospitalisation and diabetes hospitalisation compared to non-Māori in the 15 years following FEP diagnosis. Māori also appeared to have a higher adjusted risk of CVD hospitalisation than non-Māori, although this result was less precisely estimated and data were compatible with no difference. Kaplan–Meier plots show that these inequities emerge approximately 4–7 years following FEP diagnosis. Comparable M3 scores for Māori and non-Māori at FEP diagnosis further indicate that physical health inequities for Māori and non-Māori emerge following the onset on psychosis. Gender differences in outcome risk were found in supplementary analyses, which were consistent between Māori and non-Māori: males had higher risk of all-cause mortality, while females were at greater risk of diabetes, injury/poisoning, and physical health hospitalisations. In this relatively young cohort, most recorded causes of death were non-medical (330/409 deaths with a recorded cause) and ethnic differences in premature mortality were thus driven mostly by these non-medical deaths. Nearly two-thirds (63.6%) of non-medical deaths were suicide, which was the most common cause of death for Māori and non-Māori. Risk of suicide during follow-up was similar for Māori and non-Māori; however, Māori had a higher risk of accidental death than non-Māori. The leading causes of accidental death were accidental poisoning and transport accident.

Overall, follow-up risk estimates for general hospitalisation events (physical health and injury/poisoning) were much higher than for condition-specific hospitalisations (diabetes and CVD). This is unsurprising, as the former are broad categories including many common health conditions, while the latter are specific long-term conditions. However, given the long-term morbidity and mortality associated with CVD and diabetes, it is concerning to find higher risk for hospitalisation with these particular conditions among Māori experiencing psychosis at this relatively young age (31–39 years at end of follow-up). This finding is consistent with higher rates of these conditions, and associated health markers, among Māori in the general population (Ministry of Health, 2019, 2023). Moreover, sub-optimal physical health care and management in primary care may mean these conditions are more likely to progress to a hospitalisation in Māori (Cunningham et al., 2023a, 2023b; Graham and Masters-Awatere, 2020; Ministry of Health, 2019).

Consistent with previous research with young FEP cohorts (Bromet et al., 2005; Simon et al., 2018; Wiersma et al., 1998; Yuen et al., 2014), we found that suicide and accidents (in particular, accidental poisonings) accounted for approximately 80% of mortality with a recorded cause in the 15 years following FEP diagnosis. One recent systematic review reported depression, suicidality and substance use as the strongest predictors of mortality in people with schizophrenia, and thus should be prioritised for screening, support and intervention (Correll et al., 2022). However, while poor physical health is not the most immediate cause of mortality in young people experiencing psychosis, it will increase mortality later in life. In particular, inequities in CVD and diabetes admissions reported in our young cohort should be expected to progress into substantial inequities in medical-cause mortality for Māori beyond our 15-year follow-up period.

Clinicians must consider both the mental and physical health of individuals experiencing psychosis. Antipsychotic medications produce strong cardiometabolic side effects (Firth et al., 2019; Galletly et al., 2012) compounded by complex factors such as problematic substance use, lack of access to good nutrition and lower physical activity (de Leon and Diaz, 2005; Di Florio et al., 2014; Firth et al., 2019; Jackson et al., 2015; Koskinen et al., 2009; Stubbs et al., 2016; Teasdale et al., 2019; Vancampfort et al., 2017). However, despite their established cardiometabolic side effects, recent meta-analyses have shown an overall reduction in all-cause mortality for people with schizophrenia taking antipsychotic medication (Correll et al., 2022; Jia et al., 2022). Reductions in mortality are most marked for people taking second-generation antipsychotics, especially clozapine and olanzapine, which are known to carry higher cardiometabolic risks (Correll et al., 2022). Thus, it appears that effective treatment of ongoing psychosis may reduce mortality from non-medical causes, despite elevating cardiometabolic risk. Antipsychotic use is also associated with greater adherence to cardiometabolic medications in people experiencing psychosis (Solmi et al., 2022b).

As a guideline for reducing physical health inequity related to psychosis and its treatment, the Healthy Active Lives (HeAL) consensus statement (Shiers and Curtis, 2014; International Physical Health in Youth (iphYs) working group, 2013) summarises the challenges, actions and targets for improving the physical health of young people experiencing psychosis, particularly those taking antipsychotic medication. In addition to pharmaceutical and clinical treatment, there is a need to further evaluate the use of integrated care models involving support from health improvement practitioners, health coaches and support workers, alongside clinicians. Integrated care programmes aim to improve outcomes by taking a broader ‘person-centred’ approach to treatment that places an emphasis on social need (Codyre et al., 2021). In NZ, pilot evaluations of one integrated care programme, *Te Tumu Waiora*, have indicated some promising physical, mental and social outcomes, including for Māori, but further rollout and evaluation is needed (Codyre et al., 2021).

Inequities in physical health and predictors of health (e.g. socioeconomic deprivation and substance use disorders) found in this FEP cohort reflect Māori health inequities in the general population (Ministry of Health, 2018, 2019, 2023). Adverse health and social exposures for Māori are a consequence of multi-generational harm caused by settler-colonisation; this history must be navigated in pursuit of health equity (Reid et al., 2014; Waitangi Tribunal, 2021; Waitoki et al., 2014). Importantly, the NZ health system sits within this history. While most Māori report receiving good healthcare, the current health system does not meet practice guidelines for many Māori, particularly Māori experiencing psychosis (Cunningham et al., 2023a, 2023b; Espiner et al., 2021; Haitana et al., 2022a; Jansen et al., 2009). Māori experiencing psychosis may experience culturally unsafe care in the health system, including both structural and interpersonal racism (Haitana et al., 2022b, 2023; Manuel et al., 2023), and diagnostic overshadowing (Cunningham et al., 2023a, 2023b). These experiences may be improved by providing clear pathways through care, improving the clinical culture and the enhancement of *whānau* (family) involvement and support (Haitana et al., 2022a). Cultural safety for Māori is needed to improve engagement with the health system; particularly for Māori who may not view their psychosis through a colonial lens or feel comfortable with staff unable to relate to their experience (Taitimu et al., 2018). To this end, *Kaupapa* Māori healthcare and support grounded in *Kaupapa* Māori research should be available for *ranagtahi* Māori experiencing psychosis (e.g. *Meihana* model, *Hui* process and *Whānau Ora*; Haitana et al., 2020, 2022b; Huria et al., 2019; Pitama et al., 2014; Te Rau Matatini, 2015). Given these findings, effective physical and mental health care and support, facilitated by culturally safe delivery, should be prioritised for Māori immediately following FEP diagnosis.

### Future research directions

It is likely that the risks of hospitalisation and mortality reported here represent large risk increases from the same-age general population (Craig et al., 2006; Simon et al., 2018; Smith et al., 2013; Tanskanen et al., 2018; Yuen et al., 2014). People experiencing psychosis are an especially disadvantaged group with regard to physical health, and ethnic inequities we have identified for Māori sit atop this baseline inequity. However, as we did not compare the FEP cohort to the general population, we do not make these inferences on the present data. Moreover, it is difficult to compare estimated risks of diabetes and CVD hospitalisations to the general population as general population hospitalisation data for these conditions are not readily available. As a soft point of reference, the estimated rate of diabetes among 35–39-year-olds in the general population is 3.8% (Te Whatu Ora/Health New Zealand, 2024). The 15-year estimated diabetes hospitalisation risks in this study, which corresponds to approximately this age band (31–39 years old), were 6.1% for Māori and 3.3% for non-Māori. However, as these risk estimates only capture hospitalisation events, they will under-represent the actual rate of diabetes in the study cohort. It would be worthwhile for future research to compare Māori and non-Māori with FEP to the age-equivalent general population to better understand the compounding effect that psychosis and Māori ethnicity may have on physical health.

Inequities in risk of hospitalisation with diabetes and CVD at this relatively young age (at the end of follow-up, the oldest people were 39 years old) will likely contribute to mortality inequities later in life; future research is needed to analyse later mortality and health inequities. Moreover, the hospitalisation measures used in this study will not detect manageable risk factors for cardiometabolic disease (e.g. elevated lipids, HbA1c and blood pressure), which typically occur prior to hospitalisation and are also higher among Māori in the general population (Ministry of Health, 2023). There is a need to measure engagement between the health system and Māori with a diagnosis of psychosis. In particular, it would be useful to examine: primary care access, receipt of routine blood screening (lipids and HbA1c), CVD risk assessment completion, management of pre-diabetes, completion of diabetes annual reviews (for those with diabetes diagnosis), interventions made on abnormal screening results (e.g. medications such as metformin, statins) and monitoring and follow-up of abnormal screening results (e.g. follow-up screening and service contact). Considering these findings and barriers to care reported by Māori experiencing psychosis, this is an urgent research area.

### Strengths and limitations

Both PRIMHD and NMDS data were used identify a national cohort from recorded psychosis diagnoses based on standard diagnostic code sets (ICD and *DSM*). Using two identification sources reduces the probability of missing individuals meeting our inclusion criteria. Furthermore, using these data to rule out previous psychosis admissions increases our confidence that the selected index date for each cohort member is a good approximation of FEP date. It should be noted that National Collections may be subject to biases. For instance, the NHI dataset we used to code ethnicity has been estimated to undercount Māori by 16% (Harris et al., 2022), meaning some Māori will be erroneously classified as non-Māori, which may lead to underestimates of differences. We also recognise that different non-Māori ethnic groups experience varying deprivation/privilege with regard to health, healthcare access and health determinants. This study used Tiriti o Waitangi-aligned comparisons (Māori vs non-Māori); however, it is noted that health inequities for Māori experiencing psychosis would likely be higher if considered against only the dominant NZ European ethnic group.

We recognise that hospital admissions are a reasonably extreme indicator of physical ill health, and it is likely that most ill health in this age group does not result in hospitalisation. Hospitalisation outcomes (e.g. diabetes and CVD admissions) represent a conjunction of physical health needs, access to care and quality of care, rather than representing the prevalence or incidence of these health conditions in the cohort. For instance, some Māori may be considerably debilitated by a physical health condition, but not go to hospital due to barriers (Espiner et al., 2021; Graham and Masters-Awatere, 2020). It is also plausible that Māori may be more likely to present to hospital for a health condition, due to unmet need in primary health care (Harris et al., 2019; Ministry of Health, 2018, 2023). To soundly interpret these findings, it is important to recognise that the additional factors influencing hospitalisation risk (beyond the health condition itself) do systematically vary in these ways between Māori and non-Māori.

## Conclusion

Māori have higher risk of hospitalisation and mortality in the 15 years following FEP diagnosis compared to non-Māori. Suicide and accidental injuries/poisonings together caused approximately 80% of deaths with a recorded cause in the 15-year period following FEP. However, diabetes and CVD inequities found at this early life stage will lead to worse physical health and mortality outcomes later in life for Māori experiencing psychosis. Future research is needed to understand the physical health and mortality of Māori experiencing psychosis into middle and later adulthood. These findings warrant consideration of how the health system is working with Māori experiencing psychosis to prevent premature mortality and to prevent, monitor and treat cardiometabolic health conditions.

## Supplemental Material

sj-docx-1-anp-10.1177_00048674241270981 – Supplemental material for The physical health and premature mortality of Indigenous Māori following first-episode psychosis diagnosis: A 15-year follow-up study Supplemental material, sj-docx-1-anp-10.1177_00048674241270981 for The physical health and premature mortality of Indigenous Māori following first-episode psychosis diagnosis: A 15-year follow-up study by Nathan J Monk, Ruth Cunningham, James Stanley, Sue Crengle, Julie Fitzjohn, Melissa Kerdemelidis, Helen Lockett, Andre D McLachlan, Waikaremoana Waitoki and Cameron Lacey in Australian & New Zealand Journal of Psychiatry

## Notes

1. This refers only to the source of the first psychosis diagnosis for each person; it was common for people to have multiple psychosis diagnoses recorded across both PRIMHD and the NMDS.

2. Note that multiple substances may have been present; this refers only to the recorded underlying cause.

## References

[bibr1-00048674241270981] Atkinson J Salmond C Crampton P (2019) NZDep2018 Index of Deprivation: Interim Research Report, December 2019. Wellington: University of Otago.

[bibr2-00048674241270981] Ayerbe L Forgnone I Addo J , et al. (2018) Hypertension risk and clinical care in patients with bipolar disorder or schizophrenia; a systematic review and meta-analysis. Journal of Affective Disorders 225: 665–670. 28915505 10.1016/j.jad.2017.09.002

[bibr3-00048674241270981] Bromet EJ Naz B Fochtmann LJ , et al. (2005) Long-term diagnostic stability and outcome in recent first-episode cohort studies of schizophrenia. Schizophrenia Bulletin 31: 639–649. 15976012 10.1093/schbul/sbi030

[bibr4-00048674241270981] Codyre D Sharon C Didsbury L , et al. (2021) Te Tumu Waiora: The integrated primary mental health and addiction model. Available at: https://www.nzdoctor.co.nz/sites/default/files/2021-06/f36ea340-f60a-475f-aa5d-ad21ca164752.pdf (accessed 30 November 2023).

[bibr5-00048674241270981] Correll CU Solmi M Croatto G , et al. (2022) Mortality in people with schizophrenia: A systematic review and meta-analysis of relative risk and aggravating or attenuating factors. World Psychiatry 21: 248–271. 35524619 10.1002/wps.20994 PMC9077617

[bibr6-00048674241270981] Correll CU Solmi M Veronese N , et al. (2017) Prevalence, incidence and mortality from cardiovascular disease in patients with pooled and specific severe mental illness: A large-scale meta-analysis of 3,211,768 patients and 113,383,368 controls. World Psychiatry 16: 163–180. 28498599 10.1002/wps.20420 PMC5428179

[bibr7-00048674241270981] Craig TJ Ye Q Bromet EJ (2006) Mortality among first-admission patients with psychosis. Comprehensive Psychiatry 47: 246–251. 16769297 10.1016/j.comppsych.2005.11.004

[bibr8-00048674241270981] Cunningham R Imlach F Haitana T , et al. (2023a) It’s not in my head: A qualitative analysis of experiences of discrimination in people with mental health and substance use conditions seeking physical healthcare. Front Psychiatry 14: 1285431. 37908598 10.3389/fpsyt.2023.1285431 PMC10613695

[bibr9-00048674241270981] Cunningham R Imlach F Lockett H , et al. (2023b) Do patients with mental health and substance use conditions experience discrimination and diagnostic overshadowing in primary care in Aotearoa New Zealand? Results from a national online survey. Journal of Primary Health Care 15: 112–121. 37390032 10.1071/HC23015

[bibr10-00048674241270981] Cunningham R Sarfati D Peterson D , et al. (2014) Premature mortality in adults using New Zealand psychiatric services. The New Zealand Medical Journal 127: 31–41. 24929569

[bibr11-00048674241270981] Cunningham R Stanley J Haitana T , et al. (2020) The physical health of Māori with bipolar disorder. Australian and New Zealand Journal of Psychiatry 54: 1107–1114. 32929981 10.1177/0004867420954290

[bibr12-00048674241270981] Das-Munshi J Chang C-K Dutta R , et al. (2017) Ethnicity and excess mortality in severe mental illness: A cohort study. The Lancet Psychiatry 4: 389–399. 28330589 10.1016/S2215-0366(17)30097-4 PMC5406616

[bibr13-00048674241270981] Daumit GL Anthony CB Ford DE , et al. (2010) Pattern of mortality in a sample of Maryland residents with severe mental illness. Psychiatry Research 176: 242–245. 20207013 10.1016/j.psychres.2009.01.006 PMC2966471

[bibr14-00048674241270981] de Leon J Diaz FJ (2005) A meta-analysis of worldwide studies demonstrates an association between schizophrenia and tobacco smoking behaviors. Schizophrenia Research 76: 135–157. 15949648 10.1016/j.schres.2005.02.010

[bibr15-00048674241270981] Denise EJ (2014) Multiple disadvantaged statuses and health: The role of multiple forms of discrimination. Journal of Health and Social Behavior 55: 3–19. 24578393 10.1177/0022146514521215

[bibr16-00048674241270981] Di Florio A Craddock N van den Bree M (2014) Alcohol misuse in bipolar disorder. A systematic review and meta-analysis of comorbidity rates. European Psychiatry 29: 117–124. 24075633 10.1016/j.eurpsy.2013.07.004

[bibr17-00048674241270981] Dowd JJ Bengtson VL (1978) Aging in minority populations an examination of the double jeopardy hypothesis. Journal of Gerontology 33: 427–436. 748438 10.1093/geronj/33.3.427

[bibr18-00048674241270981] Espiner E Paine S-J Weston M , et al. (2021) Barriers and facilitators for Māori in accessing hospital services in Aotearoa New Zealand. The New Zealand Medical Journal 134: 47–58. 34855733

[bibr19-00048674241270981] Firth J Siddiqi N Koyanagi A , et al. (2019) The Lancet Psychiatry Commission: A blueprint for protecting physical health in people with mental illness. The Lancet Psychiatry 6: 675–712. 31324560 10.1016/S2215-0366(19)30132-4

[bibr20-00048674241270981] Fusar-Poli P Estradé A Stanghellini G , et al. (2022) The lived experience of psychosis: A bottom-up review co-written by experts by experience and academics. World Psychiatry 21: 168–188. 35524616 10.1002/wps.20959 PMC9077608

[bibr21-00048674241270981] Galletly C Castle D Dark F , et al. (2016) Royal Australian and New Zealand College of Psychiatrists clinical practice guidelines for the management of schizophrenia and related disorders. Australia and New Zealand Journal of Psychiatry 50: 410–472. 10.1177/0004867416641195 27106681

[bibr22-00048674241270981] Galletly C Foley DL Waterreus A , et al. (2012) Cardiometabolic risk factors in people with psychotic disorders: The second Australian national survey of psychosis. Australian and New Zealand Journal of Psychiatry 46: 753–761. 22761397 10.1177/0004867412453089

[bibr23-00048674241270981] Graham R Masters-Awatere B (2020) Experiences of Māori of Aotearoa New Zealand’s public health system: A systematic review of two decades of published qualitative research. Australian and New Zealand Journal of Public Health 44: 193–200. 32311187 10.1111/1753-6405.12971

[bibr24-00048674241270981] Gynther B Charlson F Obrecht K , et al. (2019) The epidemiology of psychosis in indigenous populations in Cape York and the Torres Strait. EClinicalMedicine 10: 68–77. 31193783 10.1016/j.eclinm.2019.04.009 PMC6543175

[bibr25-00048674241270981] Haitana T Pitama S Cormack D , et al. (2020) The transformative potential of Kaupapa Māori research and Indigenous methodologies: Positioning M Māori ori patient experiences of mental health services. International Journal of Qualitative Methods 19: 1609406920953752.

[bibr26-00048674241270981] Haitana T Pitama S Cormack D , et al. (2022a) Culturally competent, safe and equitable clinical care for Māori with bipolar disorder in New Zealand: The expert critique of Māori patients and Whānau. Australian and New Zealand Journal of Psychiatry 56: 648–656. 34263663 10.1177/00048674211031490 PMC9131406

[bibr27-00048674241270981] Haitana T Pitama S Cormack D , et al. (2022b) ‘If we can just dream. . .’ Māori talk about healthcare for bipolar disorder in New Zealand: A qualitative study privileging Indigenous voices on organisational transformation for health equity. The International Journal of Health Planning and Management 37: 2613–2634. 35460284 10.1002/hpm.3486 PMC9546144

[bibr28-00048674241270981] Haitana T Pitama S Cormack D , et al. (2023) ‘It absolutely needs to move out of that structure’: Māori with bipolar disorder identify structural barriers and propose solutions to reform the New Zealand mental health system. Ethnicity & Health 28: 234–256. 35040732 10.1080/13557858.2022.2027884

[bibr29-00048674241270981] Harris RB Cormack DM Stanley J (2019) Experience of racism and associations with unmet need and healthcare satisfaction: The 2011/12 adult New Zealand Health Survey. Australian and New Zealand Journal of Public Health 43: 75–80. 30296819 10.1111/1753-6405.12835

[bibr30-00048674241270981] Harris RB Paine S-J Atkinson J , et al. (2022) We still don’t count: The under-counting and under-representation of Māori in health and disability sector data. The New Zealand Medical Journal 135: 54–57. 10.26635/6965.5849 36521086

[bibr31-00048674241270981] Harris RB Stanley J Cormack DM (2018) Racism and health in New Zealand: Prevalence over time and associations between recent experience of racism and health and wellbeing measures using national survey data. PLoS ONE 13: e0196476. 10.1371/journal.pone.0196476 PMC5933753 29723240

[bibr32-00048674241270981] Health Information Standards Organisation (2017) HISO 10001:2017 Ethnicity Data Protocols. Wellington: Te Whatu Ora.

[bibr33-00048674241270981] Huria T Palmer SC Pitama S , et al. (2019) Consolidated criteria for strengthening reporting of health research involving indigenous peoples: The CONSIDER statement. BMC Medical Research Methodology 19: 173. 31399058 10.1186/s12874-019-0815-8 PMC6688310

[bibr34-00048674241270981] International Physical Health in Youth (iphYs) working group (2013) Healthy Active Lives (HeAL) consensus statement. Available at: https://www.nice.org.uk/guidance/cg178/resources/healthy-active-lives-heal-consensus-statement-international-physical-health-in-youth-working-group-pdf-191766493 (accessed 15 January 2024).

[bibr35-00048674241270981] Jackson JG Diaz FJ Lopez L , et al. (2015) A combined analysis of worldwide studies demonstrates an association between bipolar disorder and tobacco smoking behaviors in adults. Bipolar Disorders 17: 575–597. 26238269 10.1111/bdi.12319

[bibr36-00048674241270981] Jansen P Bacal K Crengle S (2009) He Ritenga Whakaaro: Māori Experiences of Health Services. Auckland: Mauri Ora Associates.

[bibr37-00048674241270981] Jia N Li Z Li X , et al. (2022) Long-term effects of antipsychotics on mortality in patients with schizophrenia: A systematic review and meta-analysis. Brazilian Journal of Psychiatry 44: 664–673. 36709510 10.47626/1516-4446-2021-2306 PMC9851750

[bibr38-00048674241270981] Jongsma HE Gayer-Anderson C Lasalvia A , et al. (2018) Treated incidence of psychotic disorders in the multinational EU-GEI study. JAMA Psychiatry 75: 36–46. 29214289 10.1001/jamapsychiatry.2017.3554 PMC5833538

[bibr39-00048674241270981] Kake TR Arnold R Ellis P (2008) Estimating the prevalence of schizophrenia among New Zealand Māori: A capture–recapture approach. Australian and New Zealand Journal of Psychiatry 42: 941–949. 18941958 10.1080/00048670802415376

[bibr40-00048674241270981] Koskinen J Löhönen J Koponen H , et al. (2009) Prevalence of alcohol use disorders in schizophrenia – A systematic review and meta-analysis. Acta Psychiatrica Scandinavica 120: 85–96. 19374633 10.1111/j.1600-0447.2009.01385.x

[bibr41-00048674241270981] Lacey C Lawrence M Paterson C , et al. (2022) Voices Forgotten or a Future of Inclusion and Equity: An Aotearoa New Zealand Perspective on Better Publication of Indigenous Mental Health Research. Thousand Oaks, CA: Sage, pp. 895–898. 10.1177/00048674221113433 35801690

[bibr42-00048674241270981] Laursen TM Nordentoft M Mortensen PB (2014) Excess early mortality in schizophrenia. Annual Review of Clinical Psychology 10: 425–448. 10.1146/annurev-clinpsy-032813-153657 24313570

[bibr43-00048674241270981] Linscott RJ Marie D Arnott KL , et al. (2006) Over-representation of Maori New Zealanders among adolescents in a schizotypy taxon. Schizophrenia Research 84: 289–296. 16542824 10.1016/j.schres.2006.02.006

[bibr44-00048674241270981] Manuel J Pitama S Clark MTR , et al. (2023) Racism, early psychosis and institutional contact: A qualitative study of Indigenous experiences. International Review of Psychiatry 35: 323–330. 37267030 10.1080/09540261.2023.2188074

[bibr45-00048674241270981] Marie D Fergusson DM Boden JM (2011) Cultural identity and pregnancy/parenthood by age 20: Evidence from a New Zealand birth cohort. Social Policy Journal of New Zealand 36: 19–36.

[bibr46-00048674241270981] Ministry of Health (2018) Tatau Kahukura: Māori Health Statistics. Wellington: Ministry of Health.

[bibr47-00048674241270981] Ministry of Health (2019) Wai 2575 Māori Health Trends Report. Wellington: Ministry of Health.

[bibr48-00048674241270981] Ministry of Health (2023) New Zealand Health Survey: Annual data explorer. Available at: https://minhealthnz.shinyapps.io/nz-health-survey-2022-23-annual-data-explorer/_w_3d73345e/#!/home (accessed 16 November 2023).

[bibr49-00048674241270981] Mitchell AJ Vancampfort D Sweers K , et al. (2011) Prevalence of metabolic syndrome and metabolic abnormalities in schizophrenia and related disorders – A systematic review and meta-analysis. Schizophrenia Bulletin 39: 306–318. 22207632 10.1093/schbul/sbr148 PMC3576174

[bibr50-00048674241270981] Moewaka Barnes H McCreanor T (2019) Colonisation, hauora and whenua in Aotearoa. Journal of the Royal Society of New Zealand 49: 19–33.

[bibr51-00048674241270981] Musuuza JS Sherman ME Knudsen KJ , et al. (2013) Analyzing excess mortality from cancer among individuals with mental illness. Cancer 119: 2469–2476. 23585241 10.1002/cncr.28091 PMC3687006

[bibr52-00048674241270981] Olfson M Gerhard T Huang C , et al. (2015) Premature mortality among adults with schizophrenia in the United States. JAMA Psychiatry 72: 1172–1181. 26509694 10.1001/jamapsychiatry.2015.1737

[bibr53-00048674241270981] Petrović-van der Deen FS Cunningham R Manuel J , et al. (2020) Exploring indigenous ethnic inequities in first episode psychosis in New Zealand – A national cohort study. Schizophrenia Research 223: 311–318. 32948382 10.1016/j.schres.2020.09.004

[bibr54-00048674241270981] Pitama S Huria T Lacey C (2014) Improving Maori health through clinical assessment: Waikare o te Waka o Meihana. The New Zealand Medical Journal 127: 107–119. 24816961

[bibr55-00048674241270981] Quigley H MacCabe JH (2019) The relationship between nicotine and psychosis. Therapeutic Advances in Psychopharmacology 9: 2045125319859969. 31308936 10.1177/2045125319859969 PMC6604123

[bibr56-00048674241270981] Rarere M Jarallah Y Kukutai T (2023) Indigenous fertility in Aotearoa New Zealand: How does ethnic identity affect birth spacing and timing? Journal of Population Research 40: 25.

[bibr57-00048674241270981] Reed SI (2008) First-episode psychosis: A literature review. International Journal of Mental Health Nursing 17: 85–91. 18307596 10.1111/j.1447-0349.2008.00515.x

[bibr58-00048674241270981] Reid J Taylor-Moore K Varona G (2014) Towards a social-structural model for understanding current disparities in Maori health and well-being. Journal of Loss and Trauma 19: 514–536.

[bibr59-00048674241270981] Reid P Cormack D Paine SJ (2019) Colonial histories, racism and health – The experience of Māori and Indigenous peoples. Public Health 172: 119–124. 31171363 10.1016/j.puhe.2019.03.027

[bibr60-00048674241270981] Schoenbaum M Sutherland JM Chappel A , et al. (2017) Twelve-month health care use and mortality in commercially insured young people with incident psychosis in the United States. Schizophrenia Bulletin 43: 1262–1272. 28398566 10.1093/schbul/sbx009 PMC5737542

[bibr61-00048674241270981] Schofield P Das-Munshi J Bécares L , et al. (2016) Minority status and mental distress: A comparison of group density effects. Psychological Medicine 46: 3051–3059. 27523979 10.1017/S0033291716001835 PMC5080664

[bibr62-00048674241270981] Shiers D Curtis J (2014) Cardiometabolic health in young people with psychosis. The Lancet Psychiatry 1: 492–494. 26361295 10.1016/S2215-0366(14)00072-8

[bibr63-00048674241270981] Simon GE Stewart C Yarborough BJ , et al. (2018) Mortality rates after the first diagnosis of psychotic disorder in adolescents and young adults. JAMA Psychiatry 75: 254–260. 29387876 10.1001/jamapsychiatry.2017.4437 PMC5885951

[bibr64-00048674241270981] Smith DJ Langan J McLean G , et al. (2013) Schizophrenia is associated with excess multiple physical-health comorbidities but low levels of recorded cardiovascular disease in primary care: Cross-sectional study. BMJ Open 3: e002808. 10.1136/bmjopen-2013-002808 PMC3641427 23599376

[bibr65-00048674241270981] Solmi M Radua J Olivola M , et al. (2022a) Age at onset of mental disorders worldwide: Large-scale meta-analysis of 192 epidemiological studies. Molecular Psychiatry 27: 281–295. 34079068 10.1038/s41380-021-01161-7 PMC8960395

[bibr66-00048674241270981] Solmi M Tiihonen J Lähteenvuo M , et al. (2022b) Antipsychotics use is associated with greater adherence to cardiometabolic medications in patients with schizophrenia: Results from a nationwide, within-subject design study. Schizophrenia Bulletin 48: 166–175. 34286338 10.1093/schbul/sbab087 PMC8781351

[bibr67-00048674241270981] Stanley J Sarfati D (2017) The new measuring multimorbidity index predicted mortality better than Charlson and Elixhauser indices among the general population. Journal of Clinical Epidemiology 92: 99–110. 28844785 10.1016/j.jclinepi.2017.08.005

[bibr68-00048674241270981] Stubbs B Williams J Gaughran F , et al. (2016) How sedentary are people with psychosis? A systematic review and meta-analysis. Schizophrenia Research 171: 103–109. 26805414 10.1016/j.schres.2016.01.034

[bibr69-00048674241270981] Taitimu M Read J McIntosh T (2018) Ngā Whakāwhitinga (standing at the crossroads): How Māori understand what Western psychiatry calls ‘schizophrenia’. Transcultural Psychiatry 55: 153–177. 29455628 10.1177/1363461518757800

[bibr70-00048674241270981] Tanskanen A Tiihonen J Taipale H (2018) Mortality in schizophrenia: 30-year nationwide follow-up study. Acta Psychiatrica Scandinavica 138: 492–499. 29900527 10.1111/acps.12913

[bibr71-00048674241270981] Te Pou o Te Whakaaro Nui (2014) Equally Well. Take Action to Improve Physical Health Outcomes for New Zealanders Who Experience Mental Illness and/or Addiction. A Consensus Position Paper. Auckland: Te Pou.

[bibr72-00048674241270981] Te Rau Matatini (2015) Kaupapa Māori Mental Health and Addiction Services: Best Practice Framework. Wellington: Te Rau Matatini.

[bibr73-00048674241270981] Te Whatu Ora/Health New Zealand (2023a) Data protection and privacy. Available at: https://www.tewhatuora.govt.nz/our-health-system/data-and-statistics/nz-health-statistics/data-protection-and-privacy/ (accessed 7 February 2024).

[bibr74-00048674241270981] Te Whatu Ora/Health New Zealand (2023b) Mortality collection. Available at: https://www.tewhatuora.govt.nz/our-health-system/data-and-statistics/nz-health-statistics/national-collections-and-surveys/collections/mortality-collection/ (accessed 12 December 2023).

[bibr75-00048674241270981] Te Whatu Ora/Health New Zealand (2023c) National Minimum Dataset (hospital events). Available at: https://www.tewhatuora.govt.nz/our-health-system/data-and-statistics/nz-health-statistics/national-collections-and-surveys/collections/national-minimum-dataset-hospital-events/ (accessed 9 November 2023).

[bibr76-00048674241270981] Te Whatu Ora/Health New Zealand (2023d) Pharmaceutical collection. Available at: https://www.tewhatuora.govt.nz/our-health-system/data-and-statistics/nz-health-statistics/national-collections-and-surveys/collections/pharmaceutical-collection/ (accessed 12 December 2023).

[bibr77-00048674241270981] Te Whatu Ora/Health New Zealand (2023e) PRIMHD – Mental health data. Available at: https://www.tewhatuora.govt.nz/our-health-system/data-and-statistics/nz-health-statistics/national-collections-and-surveys/collections/primhd-mental-health-data/ (accessed 9 November 2023).

[bibr78-00048674241270981] Te Whatu Ora/Health New Zealand (2024) Virtual diabetes register web tool. Available at: https://tewhatuora.shinyapps.io/virtual-diabetes-register-web-tool/ (accessed 14 May 2024).

[bibr79-00048674241270981] Teasdale SB Ward PB Samaras K , et al. (2019) Dietary intake of people with severe mental illness: Systematic review and meta-analysis. The British Journal of Psychiatry 214: 251–259. 30784395 10.1192/bjp.2019.20

[bibr80-00048674241270981] Termorshuizen F Smeets HM Braam AW , et al. (2014) Neighborhood ethnic density and psychotic disorders among ethnic minority groups in Utrecht City. Social Psychiatry and Psychiatric Epidemiology 49: 1093–1102. 24554124 10.1007/s00127-014-0842-z

[bibr81-00048674241270981] Vancampfort D Firth J Schuch FB , et al. (2017) Sedentary behavior and physical activity levels in people with schizophrenia, bipolar disorder and major depressive disorder: A global systematic review and meta-analysis. World Psychiatry 16: 308–315. 28941119 10.1002/wps.20458 PMC5608847

[bibr82-00048674241270981] Vandenbroucke JP Von Elm E Altman DG , et al. (2007) Strengthening the Reporting of Observational Studies in Epidemiology (STROBE): Explanation and elaboration. Annals of Internal Medicine 147: W163–W194. 10.7326/0003-4819-147-8-200710160-00010-w1 17938389

[bibr83-00048674241270981] Veling W (2013) Ethnic minority position and risk for psychotic disorders. Current Opinion in Psychiatry 26: 166–171. 23286992 10.1097/YCO.0b013e32835d9e43

[bibr84-00048674241270981] Waitangi Tribunal (2021) Hauora: Report on Stage One of the Health Services and Outcomes Kaupapa Inquiry. Wellington: Waitangi Tribunal.

[bibr85-00048674241270981] Waitoki W Nikora LW Harris PETK , et al. (2014) Māori experiences of bipolar disorder: Pathways to recovery. Auckland: Te Pou O Te Whakaaro Nui.

[bibr86-00048674241270981] Walker ER McGee RE Druss BG (2015) Mortality in mental disorders and global disease burden implications: A systematic review and meta-analysis. JAMA Psychiatry 72: 334–341. 25671328 10.1001/jamapsychiatry.2014.2502 PMC4461039

[bibr87-00048674241270981] Wiersma D Nienhuis FJ Slooff CJ , et al. (1998) Natural course of schizophrenic disorders: A 15-year followup of a Dutch incidence cohort. Schizophrenia Bulletin 24: 75–85. 9502547 10.1093/oxfordjournals.schbul.a033315

[bibr88-00048674241270981] Yuen K Harrigan SM Mackinnon AJ , et al. (2014) Long-term follow-up of all-cause and unnatural death in young people with first-episode psychosis. Schizophrenia Research 159: 70–75. 25151199 10.1016/j.schres.2014.07.042

[bibr89-00048674241270981] Yung NCL Wong CSM Chan JKN , et al. (2023) Mortality rates in people with first diagnosis of schizophrenia-spectrum disorders: A 5-year population-based cohort study. Australian and New Zealand Journal of Psychiatry 57: 854–864. 36062474 10.1177/00048674221121575

