# Supplementary material for: The physical health and premature mortality of Indigenous Māori following first-episode psychosis diagnosis: A 15-year follow-up study
Source: Aust N Z J Psychiatry. 2024 Aug 21;58(11):963–76. doi: 10.1177/00048674241270981 (PMC11529131; doi:10.1177/00048674241270981)
Supplement: sj-docx-1-anp-10.1177_00048674241270981 – Supplemental material for The physical health and premature mortality of Indigenous Māori following first-episode psychosis diagnosis: A 15-year follow-up study [file sj-docx-1-anp-10.1177_00048674241270981.docx]

**Supplementary materials**

**The physical health and mortality of Indigenous Māori following first episode psychosis diagnosis: A 15-year follow-up study**

Nathan J. Monk, Ruth Cunningham, James Stanley, Sue Crengle, Julie Fitzjohn, Melissa Kerdemelidis, Helen Lockett, Andre D. McLachlan, Waikaremoana Waitoki, Cameron Lacey

| Table S1. Diagnostic codes for psychotic disorders | | |
| --- | --- | --- |
| Psychotic disorder | ICD-9/DSM-IV | ICD-10 |
| Schizophrenia | 29510-29569, 29580-29595 | F20 |
| Bipolar I | 29600-29602, 29604-29606, 29640-29642, 29644-29646, 29650-29652, 29654-29656, 29660-29662, 29664-29666, 2967, 29680, 29689 | F302-F309, F312, F313, F315, F316 |
| Schizoaffective | 29570-29575 | F25 |
| Depression with psychosis | 29624, 29634 | F323, F333 |
| Substance with psychosis | 2913-2915, 29211-29212 | F105, F115, F125, F135, F145, F155, F165, F175, F185, F195 |
| Other | 2988, 2971, 2973 | F22-F24, F28 |
| Organic | 29381, 29382 | F060, F062 |
| Not otherwise specified | 2989 | F29 |
|  | | |

| Table S2. Diagnostic codes for cardiovascular disease (CVD) | | |
| --- | --- | --- |
| CVD condition | ICD-9 | ICD-10 |
| Myocardial infarction | 410 | I210-I214, I219-I221, I228, I229 |
| Unstable angina | - | I200 |
| Other coronary heart disease | 41189, 4148, 4275, 4296, 4139, 4131, 4295, 4148, 41410, 41419, 412, 42971, 42979, 41411, 41412, 41181 | I236, I248, I249, I255, I46, I235, I209, I201, I234, I256, I253, I252, I232, I230, I254, I233, I208, I240, I238, I231 |
| Ischaemic stroke | 433, 434, 436 | I63, I64 |
| Haemorrhagic stroke | 430, 431, 432 | I60, I61 |
| Transient ischaemic attack | 4350, 4351, 4352, 4353, 4358, 4359, 4378, 36234 | G450, G451, G452, G453, G458, G459, G46 |
| Peripheral vascular disease | 25070, 25071, 25072, 25073, 44381, 4410, 4411, 4413, 4415, 4416, 4439, 444 | E1050, E1051, E1052, E1150, E1151, E1152, E1451, E1452, I7021, I7022, I7023, I7024, I710, I711, I713, I715, I718, I739, I74 |
| Congestive heart failure | 40201, 40211, 40291, 40401, 40411, 40491, 40403, 40413, 40493, 428 | I110, I130, I132, I50 |
| Other ischaemic CVD-related codes | 41400, 41401, 4141, 4142, 4143, 4144, 4148, 4149, 4275, 44329, 4370, 438, 4400, 4401, 4402, 4404, 4408, 4409, 441, V4581, V4582, V4502, V4321, V4322, V4509, V4500 | E1059, E1159, E1459, I250, I251, I258, I259, I46, I65, I66, I670, I672, I690, I691, I693, I694, I698, I700, I701, I702, I708, I709, I714, Z951, Z955, Z958, Z959 |

| Table S3. Gender comparisons of outcome risks for Māori and non-Māori | | | | |
| --- | --- | --- | --- | --- |
|  | Māori | | Non-Māori | |
|  | Crude HR (95% CI) | Adjusted HR (95% CI) | Crude HR (95% CI) | Adjusted HR (95% CI) |
| Mortality, all cause^b^ | 1.45 (1.07, 1.96) | 1.44 (1.07 1.95) | 1.58 (1.23, 2.03) | 1.56 (1.21, 2.00) |
| Hospitalisation, physical health | 0.60 (0.55, 0.65) | 0.60 (0.55, 0.65) | 0.61 (0.57, 0.65) | 0.61 (0.57, 0.65) |
| Hospitalisation, injury/poisoning | 0.72 (0.66, 0.78) | 0.72 (0.66, 0.78) | 0.74 (0.70, 0.79) | 0.74 (0.70, 0.79) |
| Hospitalisation, diabetes | 0.48 (0.35, 0.65) | 0.48 (0.35, 0.65) | 0.64 (0.48, 0.85) | 0.61 (0.46, 0.81) |
| Hospitalisation, CVD | 1.48 (0.87, 2.50) | 1.33 (0.92, 1.92) | 0.88 (0.58, 1.34) | 0.88 (0.58, 1.34) |
| Notes: HR >1 indicates males at greater risk; Adjusted for age and socioeconomic deprivation; Cause-specific deaths not tested due to small number of cases when sub-sampling. | | | | |


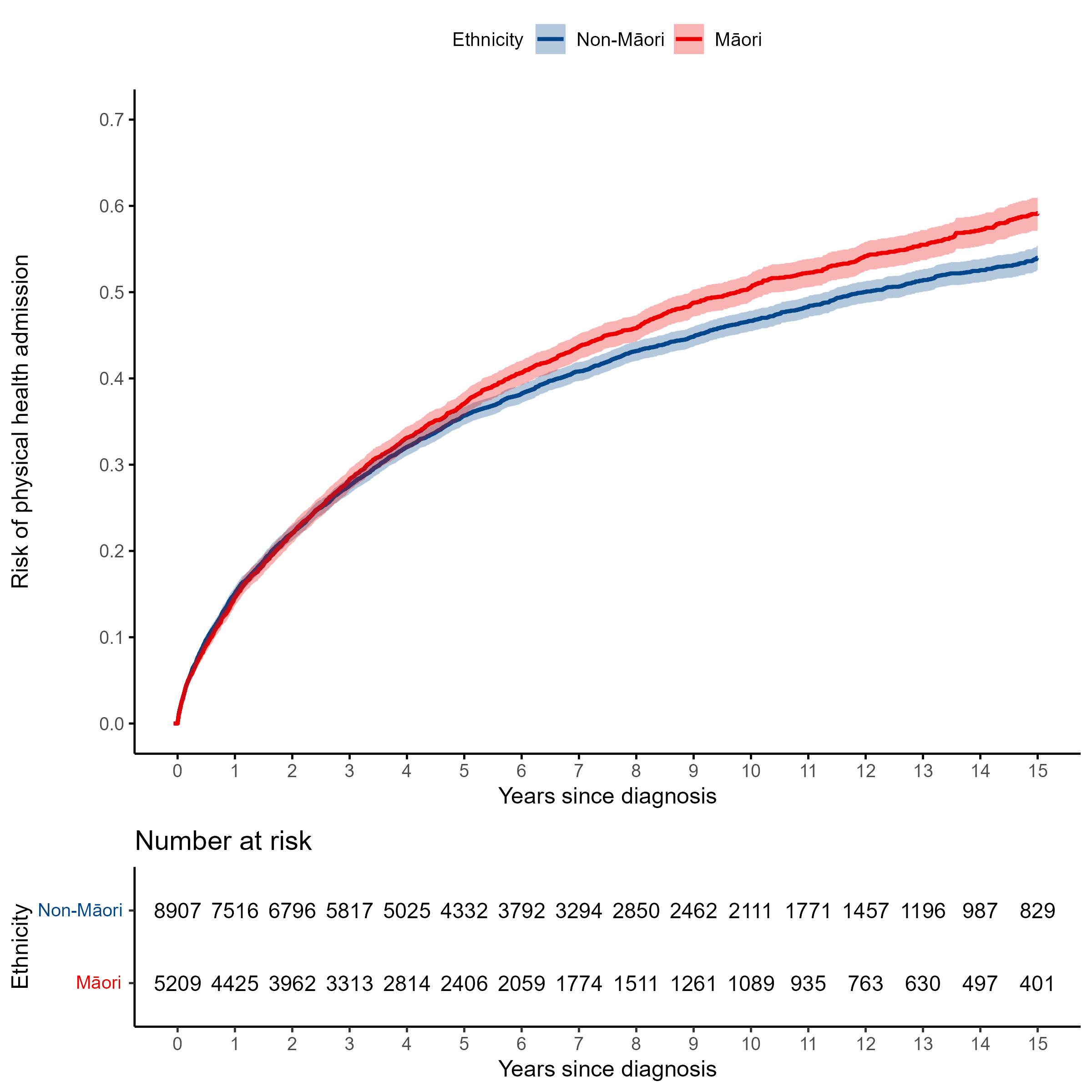


Figure S1. Māori and non-Māori Kaplan-Meier plots of physical health hospitalisation risk in 15-year follow-up from first episode psychosis (shaded areas indicate 95% confidence intervals)


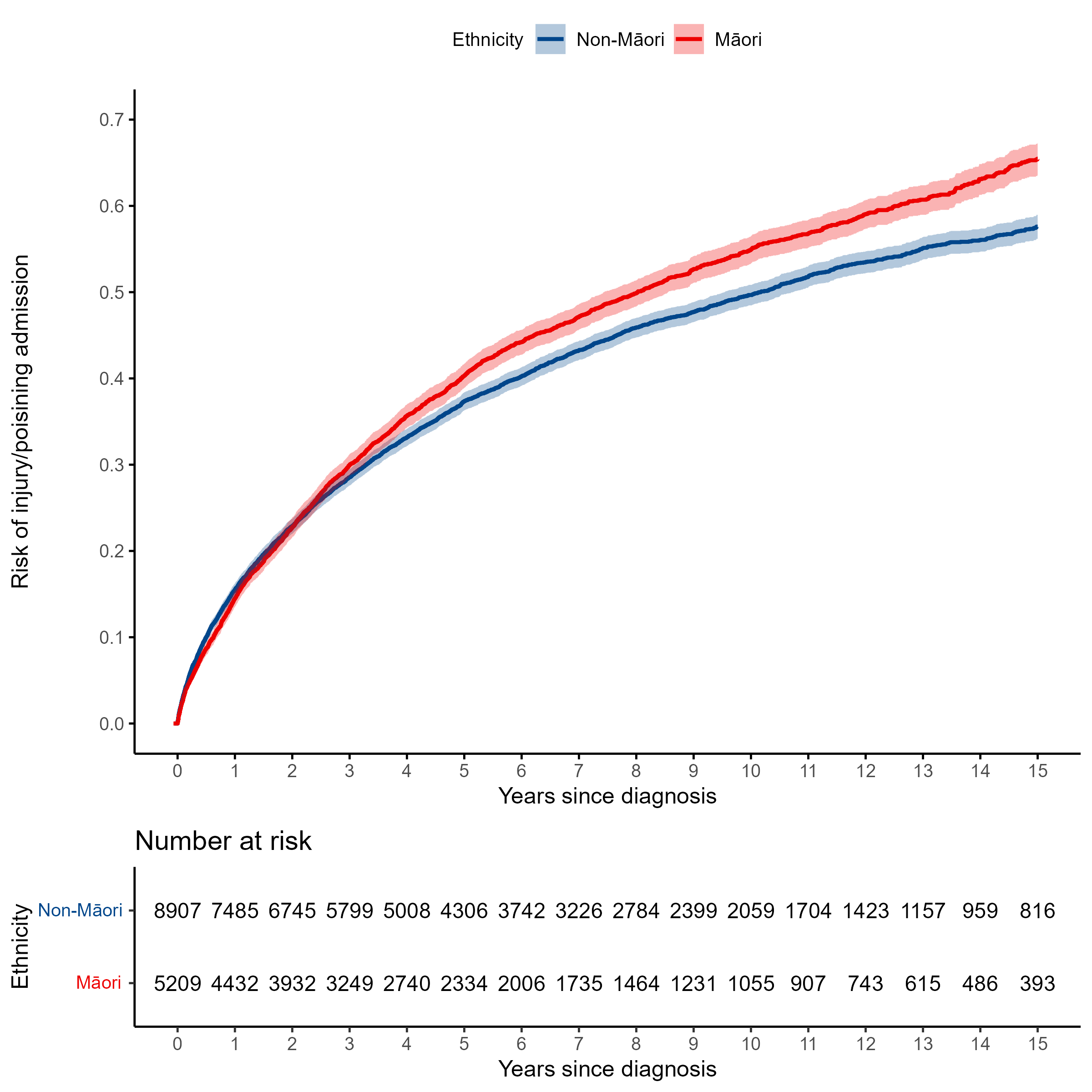


Figure S2. Māori and non-Māori Kaplan-Meier plots of injury/poisoning hospitalisation risk in 15-year follow-up from first episode psychosis (shaded areas indicate 95% confidence intervals)


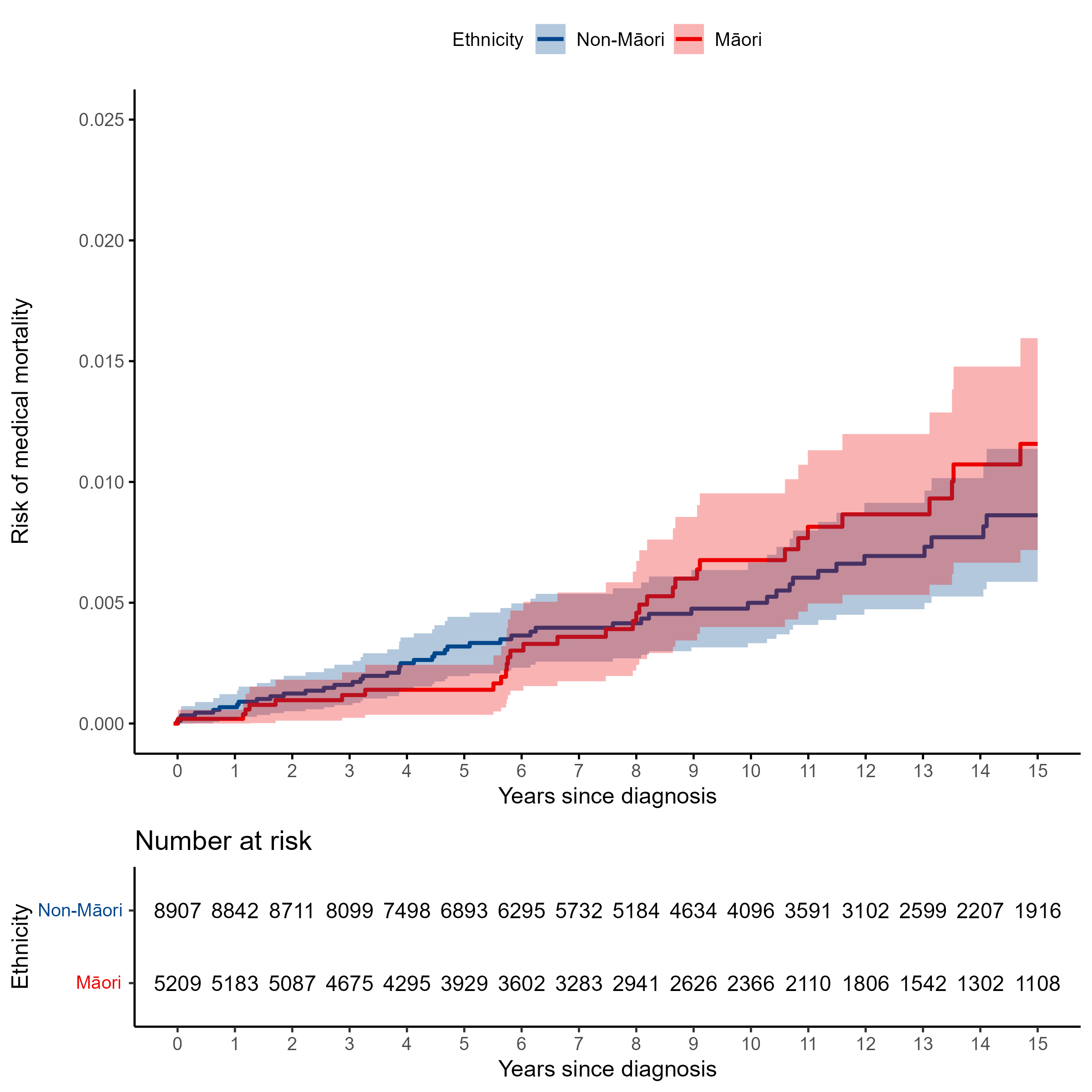


Figure S3. Māori and non-Māori Kaplan-Meier plots of medical mortality risk in 15-year follow-up from first episode psychosis (shaded areas indicate 95% confidence intervals)


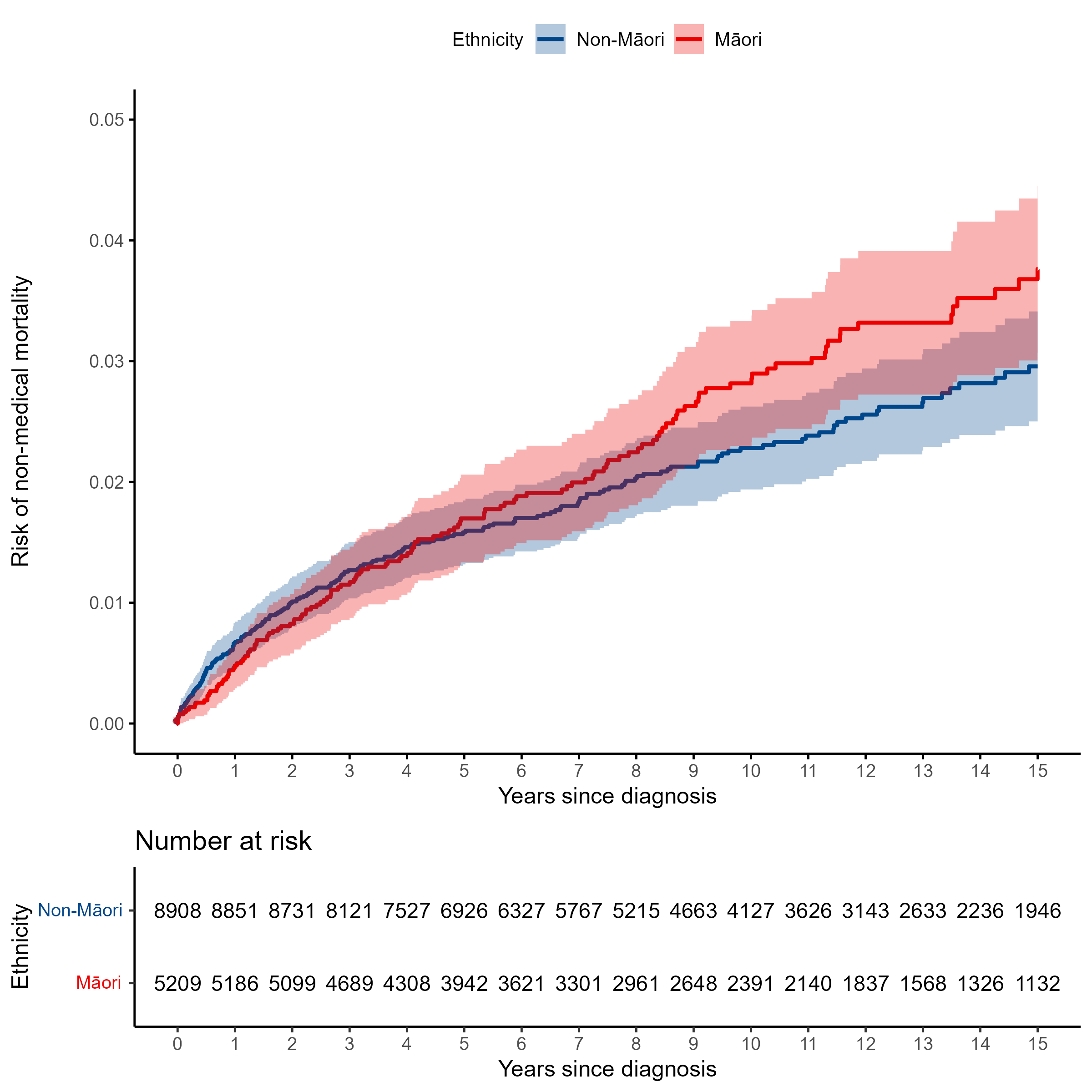


Figure S4. Māori and non-Māori Kaplan-Meier plots of non-medical mortality risk in 15-year follow-up from first episode psychosis (shaded areas indicate 95% confidence intervals)
